# Strengthening medical training programmes by focusing on professional transitions: a national bridging programme to prepare medical school graduates for their role as medical interns in Botswana

**DOI:** 10.1186/s12909-017-1102-1

**Published:** 2017-12-21

**Authors:** Michael J. Peluso, Rebecca Luckett, Savara Mantzor, Alemayhu G. Bedada, Paul Saleeb, Miriam Haverkamp, Mosepele Mosepele, Cecil Haverkamp, Rosa Maoto, Detlef Prozesky, Neo Tapela, Oathokwa Nkomazana, Tomer Barak

**Affiliations:** 1 Botswana-Harvard Partnership, Gaborone, Botswana; 2 Department of Medicine and Division of Global Health Equity, Brigham and Women’s Hospital, Boston, MA USA; 3 Harvard Medical School, Boston, USA; 4 Department of Obstetrics and Gynecology, Scottish Livingstone Hospital, Molepolole, Botswana; 5 Department of Obstetrics and Gynecology, Beth Israel Deaconess Medical Center, Boston, USA; 6 Children’s Hospital of Philadelphia, Philadelphia, USA; 7 Department of Paediatrics, Princess Marina Hospital, Gaborone, Botswana; 8 Botswana-UPenn Partnership, Gaborone, Botswana; 9 Department of Surgery, Faculty of Medicine, University of Botswana, Gaborone, Botswana; 10 University of Maryland, College Park, MD USA; 11 Botswana-University of Maryland School of Medicine Health Initiative, Gaborone, Botswana; 12 Department of Medicine, Princess Marina Hospital, Gaborone, Botswana; 13 University Research Co., Gaborone, Botswana; 14 Medical Internship Training Programme, Gaborone, Botswana; 15 Department of Medical Education, University of Botswana, Gaborone, Botswana; 16 Botswana Ministry of Health, Gaborone, Botswana; 17 Faculty of Medicine, University of Botswana, Gaborone, Botswana; 18 Department of Medicine, Scottish Livingstone Hospital, Molepolole, Botswana; 19 Department of Medicine, Beth Israel Deaconess Medical Center, Boston, USA

**Keywords:** Graduate medical education, Global health, Sub-Saharan Africa, Health workforce, Capacity building

## Abstract

**Background:**

The improvement of existing medical training programmes in resource-constrained settings is seen as key to addressing the challenge of retaining medical graduates trained at considerable cost both in-country and abroad. In Botswana, the establishment of the national Medical Internship Training Programme (MIT) in 2014 was a first step in efforts to promote retention through the expansion and standardization of internship training, but MIT faces a major challenge related to variability between incoming trainees due to factors such as their completion of undergraduate medical training in different settings. To address this challenge, in August 2016 we piloted a bridging programme for foreign and locally trained medical graduates that aimed to facilitate their transition into internship training. This study aimed to describe the programme and evaluate its impact on the participants’ self-rated perceptions of their knowledge, experience, clinical skills, and familiarity with Botswana’s healthcare system.

**Methods:**

We conducted a national, intensive, two-week programme designed to facilitate the transition from medical student to intern and to prepare all incoming interns for their work in Botswana’s health system. Participants included all interns entering in August 2016. Formats included lectures, workshops, simulations, discussions, and reflection-oriented activities. The Kellogg Foundation Outcomes Logic Model was used to evaluate the programme, and participants self-rated their knowledge, skills, and attitudes across each of the programme objectives on paired questionnaires before and after participation.

**Results:**

48/54 participants (89%) provided paired data. Participants reported a high degree of satisfaction with the programme (mean 4.2/5). Self-rated preparedness improved after participation (mean 3.2 versus 3.7, *p* < 0.001), as did confidence across 18/19 knowledge/skill domains, suggesting that participants felt that the programme prepared them for their internship training. Exploratory analysis revealed that 20/25 participants (80%) reporting either no effect or a negative effect following participation had rated themselves “extremely” or “quite” prepared beforehand, suggesting the programme grounded expectations for interns who initially were overconfident. In contrast, no interns who had initially rated themselves “moderately” or “somewhat” prepared reported a decline in their self-rated sense of preparedness. Interns commented on the benefits of learning about roles/responsibilities, interacting with clinicians from Botswana’s healthcare sectors, and the sense of community the programme engendered.

**Conclusions:**

This programme was feasible to implement and was well-received by participants. Overall, participants perceived an enhancement of their knowledge, skills, and expectations about their role in Botswana’s health system after completion of the programme. Our results are likely to be of interest to educators dedicated to training, professional transitions, and career pathways in similar settings in the region and beyond.

**Electronic supplementary material:**

The online version of this article (10.1186/s12909-017-1102-1) contains supplementary material, which is available to authorized users.

## Background

The quality of medical school and postgraduate training of doctors is a top priority for the delivery of high-quality healthcare within constrained systems in settings like sub-Saharan Africa [1–5]. While internship training is a precursor for higher-level qualifications in some settings, in many countries it remains the concluding step before licensure and independent practice [1–4, 6–10]. The well-documented mismatch between the health burden and the healthcare workforce in sub-Saharan Africa has historically led to an emphasis on the quantity, but not necessarily the quality, of medical training opportunities [4–6, 11, 12]. These factors combine with limited opportunities for continuing education and career advancement to fuel doctor migration and make it challenging for African countries to retain trained medical graduates and deliver high-quality care [7, 10, 13, 14].

In Botswana, a middle-income country of approximately two million inhabitants in southern Africa, only a small proportion of doctors are trained as specialists and the bulk of medical care is provided by doctors (referred to as “medical officers”) who have completed medical school and a one-year post-graduate internship [6]. Most doctors in Botswana practice in a public healthcare system, which is constrained by a shortage of providers and limited resources, and faces widespread epidemics of infectious and non-communicable diseases [2, 4, 6, 11, 15–19]. In this setting, internship training is intended, over a short period of time, to produce doctors competent to practice safely and independently across a wide scope of medical, pediatric, surgical, and obstetric clinical areas.

Internship training in Botswana had previously taken place in the country’s two urban referral hospitals and was guided by a list of required competencies put forth by the Botswana Health Professions Council. It lacked a standardized format, standardized content, and formal supervision structure. The national Medical Internship Training Programme (MIT) was launched in 2014 to provide a sustainable framework for quality internship training as a shared institutional responsibility of three main stakeholders: the Ministry of the Health, the Botswana Health Professions Council, and the University of Botswana Faculty of Medicine [16, 20–23]. MIT aims to optimize the transition of recently graduated medical doctors to their roles as fully autonomous medical practitioners through ensuring quality training that would prepare them to practice in Botswana’s public health system, particularly in remote and rural areas. To this end MIT has expanded training to eight sites across the country including rural district hospitals and worked to standardize the format and content of internship training through formally supervised practice in internal medicine, pediatrics, obstetrics and gynecology, and surgery. In addition, by demonstrating an investment in medical graduates’ professional development and providing an opportunity for high-quality training in-country, MIT hopes to contribute to the wider national effort of promoting the retention of medical doctors.

Under MIT, internship training in Botswana continues to face several challenges leading to variability in the level of knowledge, skills, and clinical competence of graduates. While a growing number of interns are graduates of Botswana’s single medical school [16], a significant proportion (approximately 30–40%) are citizens of Botswana who attended medical school abroad – in South Africa, Australia, Western and Eastern Europe, the U.S., and the Caribbean. As such, their knowledge and skills reflect the medical cultures, traditions, and system settings in which they studied, leading to significant baseline variability among participants as they embark upon internship training. This includes, but is not limited to, familiarity with the local health system and context, practical procedural skills and previous exposure to medical subspecialties, advanced medical technologies, primary health care, and rural medicine. Upon registration in Botswana, graduates of in-country and foreign medical schools are assigned to eight sites throughout the country, which differ in facility level (including district and referral hospitals), clinical resources, availability of clinical educators, and access to specialist consultants. In addition, training sites tend to vary in terms of the orientation, preparation, and expectations of trainees. After 1 year, graduates are posted as medical officers across the country, often with limited access to supervision by more experienced clinicians or opportunities for continuing education.

The variability in medical graduates entering the MIT in the setting of the well-described challenges associated with the transition of students to doctors-in-training globally [24] was recognized as an area for intervention to improve outcomes of internship training across sites. In collaboration with the Ministry of Health, the Botswana Health Professions Council, and the University of Botswana, we designed, implemented, and evaluated an intensive, two-week Pre-Boarding Internship Boot Camp and Bridging Programme for foreign and locally trained medical graduates. In this article, we report on the programme’s structure and content, and discuss its impact on the first group of trainees’ self-rated perceptions of their medical knowledge, skills, and preparedness for internship training.

## Methods

### Programme design and implementation

The Pre-Boarding Internship Boot Camp and Bridging Programme was designed with the goal of contributing to the effective training of a new cohort of safe, competent, and committed medical doctors in Botswana. The objectives of the programme are listed in Table 1.

**Table 1:** Aims and objectives of the Pre-Boarding Boot Camp and Bridging Programme

Prepare incoming interns for a successful transition into their internship programme – thereby ensuring intended learning objectives are achieved from the very first day of internship.
Ensure a common basis and level of pre-existing knowledge and skills of the entire cohort of interns, whether trained locally or foreign graduates.
Provide interns with orientation and relevant contextual information with regard to the organization of the Botswana health and medical system, and their responsibilities and roles as members of the medical profession.
Create opportunities for professional networking among peers, with supervisors and other key personnel under the Botswana Medical Internship Training programme.
Engage the country’s future doctors and healthcare leaders in an active discussion about the future of healthcare in Botswana and their role within it.

The programme format and contents were selected with a view towards providing a high-paced, high-yield, interactive training experience that would reinforce essential practical clinical and professional skills as well as provide a comprehensive orientation to internship training in Botswana, the local health system, and context and interns’ role within it. The programme was organized into three modules, running concurrently over 2 weeks. Table 2 describes the modules and the core content included in each, and the full schedule is provided in the Additional file 1. Each module was composed of a combination of didactic teaching, clinical skills workshops/simulations, group discussions, panel discussions, and reflection-oriented activities.

**Table 2:** Modules and core content of the Pre-Boarding Boot Camp and Bridging Programme

Module 1: The Botswana Healthcare Context
Internship rules and regulations
MIT key parameters, public service expectations, on-call regulations, work hour regulations, supervision regulations
Interns’ roles and responsibilities
Service related standards and expectations
The health system in Botswana
Guide to the healthcare system, primary healthcare in Botswana, perspectives from the Ministry of Health
The socio-economic context of healthcare in Botswana
Perspectives from the community
Bridging programme for foreign graduates
Hospitals in Botswana, tuberculosis in Botswana, referral systems, transitional challenges
International perspectives for University of Botswana graduates
Medical schools and internship programmes, Botswana healthcare in an international context, the South African healthcare system
Module 2: Clinical Knowledge and Skills
Internal medicine emergencies workshop
Septic shock, hyperglycemic emergencies, unresponsive patient, chest pain, seizure, hypertensive crisis, potassium disturbances, shortness of breath
HIV care provider training workshop
AIDS Clinical Care Fundamentals 2016 Course
Pediatric emergencies workshop
Assessment of the critically ill child, respiratory distress, status epilepticus, shock, neonatal hypoglycemia, management of “flat” neonate, neonatal seizures, intraosseous line placement, paediatric intubation, neonatal resuscitation
Obstetric emergencies workshop
Bleeding in early pregnancy, antepartum hemorrhage, postpartum hemorrhage, labor management, hypertensive disease, septic shock
Surgical skills workshop
Management of epistaxis, polytrauma, plaster application, evaluation and management of surgical wounds, suturing, knot tying
Module 3: Becoming a Well-Rounded Clinician
Professionalism workshop
Introduction to professionalism, delivering bad news, managing “difficult” patients and families with compassion and understanding, coping in the workplace, hierarchy and whistle-blowing, transformative action and advocating positive change
Intern perspectives
Experience, thoughts, and advice from recently graduated interns
Resources and technology
Utilizing technological resources to access guidelines, clinical references, and the medical literature
Career paths
Career pathways in Botswana

A planning committee, which included representatives of MIT stakeholders, partner organizations, and internship training sites, was responsible for programme design and selection of contents and formats based upon the programme’s objectives and available human and material resources. Clinicians and other experts from across the nation’s healthcare sector volunteered their time to lead programme activities. They participated in one of three roles: module director, workshop director, or programme faculty. Module directors were responsible for the organization and implementation of each module. They recruited workshop leaders and programme faculty. Workshop leaders worked with module directors to coordinate and implement each of the skills workshops/simulation sessions, which often involved multiple programme faculty teaching in small groups. Finally, programme faculty participated in a teaching, lecturing, or panelist role in one of the many programme sessions. A faculty coordinator oversaw the entire programme to ensure that all topic areas were covered with appropriate overlap, and an administrative coordinator managed programme logistics.

All incoming interns accepted for the August 2016 intake of the MIT were required to participate. The MIT programme secretariat provided administrative support and covered costs associated with the logistics of the programme (including, but not limited to, provision of temporary housing for all interns, venue rental for programme activities, and provision of food and programme materials).

The programme was made possible by a collaboration between the immediate MIT stakeholders (Ministry of Health, Botswana Health Professions Council, University of Botswana Faculty of Medicine), and its international partners. These included the Botswana-Harvard Partnership, University of Pennsylvania, University of Maryland, World Health Organization, and the University Research Co. (URC), reflecting the long tradition of effective international partnership in the Botswana health sector and around the national HIV response.

### Programme evaluation

We used the W.K. Kellogg Foundation Outcomes Logic Model for Programme Planning to implement and evaluate the programme [25]. Pre-determined outputs included attendance, participant demographics, and satisfaction with the programme and its components. Pre-determined outcomes and impact included interns’ self-reported preparedness for intern year across a number of clinical domains, as well as confidence related to specific clinical skills. In order to evaluate output and outcomes, we administered pre- and post-programme surveys, which asked participants to rate their preparedness and confidence across multiple domains using 5-point Likert scales, developed in accordance with recommended guidelines [26, 27]. These surveys were linked using individually generated, anonymous identifiers that allowed us to match participants’ scores across timepoints. In addition, participants were asked to complete anonymous feedback forms on each of the five workshops.

Data were collected on paper forms and input into an electronic database by a single individual. 10% of entries were reviewed for accuracy. Analyses utilized descriptive statistics, paired sample t-tests, and z-tests of sample proportions and were conducted using SPSS version 24.0 and GraphPad Prism version 5.0d. Where appropriate, Likert responses were analyzed and interpreted quantitatively [28]. Two reviewers used content analysis to identify and reconcile themes from the free-response questions [29, 30]. The project received ethical approval from the Botswana Health Resources Development Council and the Beth-Israel Deaconess Medical Center Committee on Clinical Investigations as part of a larger study of the internship programme.

## Results

### Characteristics of participants

53/54 individuals (98%) submitted the pre-programme survey and 51 (94%) completed the post-programme survey; 48 respondents (89%) provided participant-generated identifying codes at both timepoints to allow for data matching. Table 3 describes the demographics of the pre-survey respondents.

**Table 3:** Demographic characteristics of 53 programme participants for whom pre-programme data were available

Characteristic	n (%)
Gender	
Male	28 (53%)
Female	25 (47%)
Age	
21–24 years	17 (32%)
25–29 years	32 (60%)
30+ years	4 (8%)
Medical School	
University of Botswana	37 (70%)
Outside Botswana	16 (30%)

### Pre-programme enthusiasm and preparedness

**Fig. 1:**
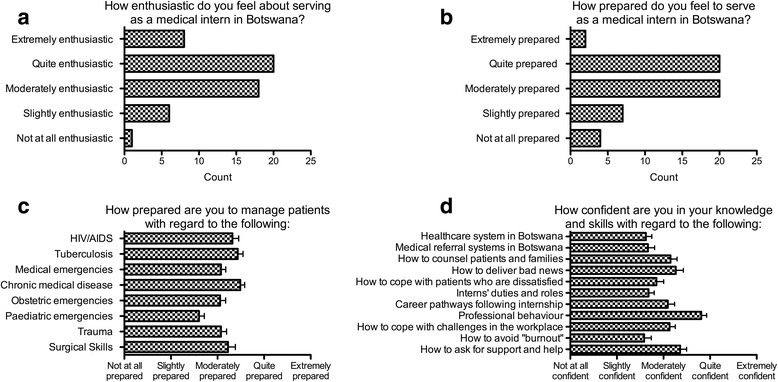
**a**-**d**. Self-reported level of enthusiasm, preparedness, and confidence of 53 participants prior to participation in the programme. **a** Self-reported level of enthusiasm. **b** Self-reported level of preparedness. **c** Self-reported level of preparedness across core clinical domains. **d** Self-reported level of confidence across knowledge and skill domains

Figure 1 shows the pre-programme responses of 53 individuals. Before participation in the programme, interns reported moderate degrees of enthusiasm (mean score 3.5 on a 5-point scale) and self-rated preparedness (mean score 3.2 on a 5-point scale) for serving in their role as medical interns in Botswana (Fig. 1a-b). They also reported being moderately prepared across clinical domains and slightly to moderately confident across knowledge and skill domains (Fig. 1c-d).

### Satisfaction and effectiveness

**Fig. 2:**
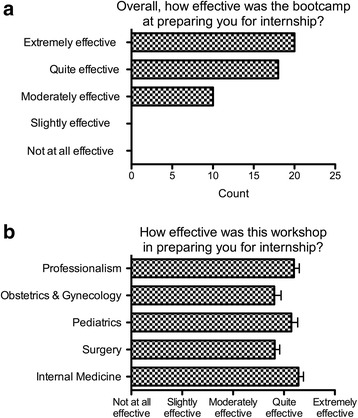
**a**-**b**. Post-programme responses of 51 participants regarding overall effectiveness of the programme and the effectiveness of individual programme workshops. **a** Perceived level of effectiveness of overall programme. **b** Perceived level of effectiveness of individual workshops

Overall, interns reported a high degree of satisfaction with the programme, rating it with a mean score of 4.2 on the 5-point scale (range, 3–5; Fig. 2a). They felt that the workshops were effective in preparing them for internship (Fig. 2b), and that the materials were effective for each of the workshops (data not shown).

### Programme preparedness and confidence outcomes

**Fig. 3:**
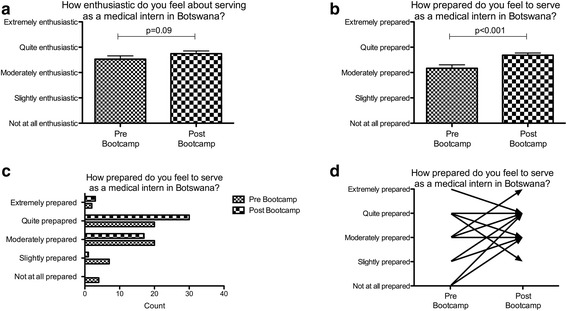
Paired analyses of enthusiasm and preparedness of 48 participants. **a** Self-perceived level of enthusiasm before and after participation in the programme. **b** Self-reported level of preparedness before and after participation, aggregated. **c** Self- reported level of preparedness before and after participation, stratified by response. **d** Directionality and effect size of reported change within individuals before and after participation

Interns remained moderately enthusiastic following participation in the training (mean score 3.5 versus 3.7, *p* = 0.09; Fig. 3a). The paired analysis showed an improvement in self-rated level of preparedness after participation in the programme, although the effect size was modest (mean score 3.2 versus 3.7, *p* < 0.001; Fig. 3b). The data distribution (Fig. 3c) showed that more respondents indicated that they felt “extremely prepared” or “quite prepared” than prior to participation, and fewer respondents selected “moderately prepared” or “slightly prepared” after participation. Four respondents had indicated that they felt “not at all prepared” prior to participation; no respondents selected “not at all prepared” after the programme.

Potential explanations for the modest effect size on self-reported preparedness were explored in an individual level analysis, shown in Fig. 3d, which maps the directionality and degree of change in each individual participant based upon his or her starting point of self-assessed preparedness. Of 48 pairs, 23 (48%) reported a positive change, 19 (40%) reported no change, and 6 (13%) reported a negative change. Of the 19 participants that reported no change, 14 (74%) reported feeling “quite prepared” both before and after participation, while 5 (26%) had reported feeling “moderately prepared” at both time points. Of the 6 participants with a negative change, all had reported feeling “extremely prepared” or “quite prepared” prior to participation in the programme.

**Fig. 4:**
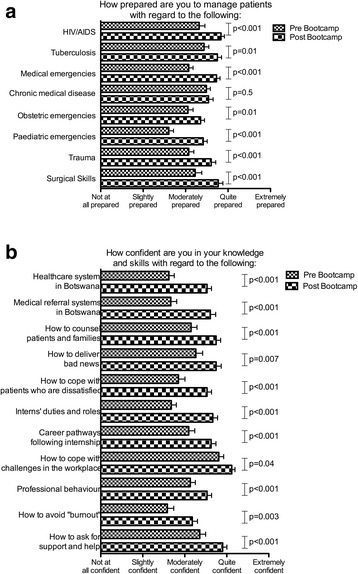
Paired analyses of 48 participants’ self-ratings on various clinical, knowledge, and skill domains. **a** Self- reported level of preparedness across clinical domains. **b** Self- reported level of confidence across knowledge and skill domains

Following participation, interns felt more prepared across all clinical domains that had been covered in the programme (Fig. 4a). They did not improve in the domain that was assessed but not included in the programme’s training content (management of chronic medical disease). They also demonstrated improvement in confidence across all knowledge and skill areas covered in the programme (Fig. 4b).

### Exploratory analyses stratified by medical school

While our sample was small, paired data were available for 34 graduates of the University of Botswana and 14 graduates of medical schools outside Botswana. There were no differences in pre- or post-programme scores for overall preparedness. 16/37 (43%) graduates of Botswana’s medical school rated themselves as “quite prepared” or “extremely prepared” prior to the programme, compared with 6/16 (38%) graduates of foreign medical schools (*p* = 0.73); after the intervention, these proportions were 24/34 (74%) and 7/14 (50%), respectively (*p* = 0.12). When the direction of individual scores were stratified by medical school, 15/34 (44%) University of Botswana (UB) graduates and 3/14 (21%) foreign graduates reported no change (*p* = 0.13), 16/34 (47%) UB graduates and 8/14 (57%) foreign graduates reported a change in a positive direction (*p* = 0.53), and 3/34 (9%) of UB graduates and 3/14 (21%) of foreign graduates reported a change in a negative direction (*p* = 0.25).

### Free-response data

Participants were given the opportunity to answer free response questions before and after participation in the programme. Prior to the programme, the following themes were notable as causes of anxiety or concern with regard to starting internship training: managing emergencies, managing the logistics of internship, uncertainty regarding roles and responsibilities of an intern, quality of training, workload/work environment, making medical errors, practicing independently, and medical knowledge.

When asked about what they found most useful about the programme, participants commented on a variety of items including the interactive small-group case discussions, interactive skills and simulation sessions, HIV and tuberculosis training, professionalism training, and knowledge review. They also commented on the sense of community and camaraderie they felt as a result of the programme, including meeting new colleagues, interacting with more senior clinicians from a variety of internship training sites, learning more about the roles and responsibilities of an intern, getting career advice, smoothing the transition between student and doctor, and “bridging the gap” between local and foreign trained medical graduates. Representative comments included:

“It will help us to survive internship with the knowledge we gained [about] how to act in difficult situations.”

“[While it] prepared us for work, it also helped us to know each other and meet our colleagues.”

“Bridging the gap by giving essential tools to make the transition easier. I am less anxious than at the start.”

“It helped remind me and even teach me about a lot of…conditions that would most likely be there on my first call.”

When asked what suggestions they had to improve the programme for the future, participant responses included recommendations to include additional specific fields of practice (e.g. radiology) or specific topics (e.g. croup, heart failure), modify the timing/duration of the programme (e.g., longer programme duration with shorter days), consistently provide hard-copy training materials, and include more small group discussions and case-based format sessions.

## Discussion

The Pre-Boarding Boot Camp and Bridging Programme aimed to prepare a diverse group of medical school graduates to navigate the transition between their previous role as medical students and their new role as medical interns in Botswana. The programme had a meaningful impact on interns’ self-rated perception of preparedness and self-rated perception of confidence in their knowledge and skills across a variety of training domains. We believe that pre-internship training programmes like this one may be of benefit elsewhere and that the goals, objectives, format, and content as presented here may be replicated and implemented with only minor adjustments in other countries facing similar challenges in the region and beyond.

### High level of pre-programme preparedness and confidence

One notable result was the relatively high level of preparedness and confidence at which participants rated themselves prior to the programme. While this sense of preparedness suggests that medical school graduates feel ready for supervised practice, the relatively lower sense of preparedness and confidence across specific clinical, knowledge, and skill domains suggests that their general sense of preparedness may have been inflated. While our study was not powered to make distinctions between graduates of different medical schools, further investigation into the origins and effects of these findings is warranted.

### Recognizing limitations to knowledge and skill

One explanation for the relatively small effect size in the improvement in medical graduates’ sense of preparedness for internship training is evident in the individual level analysis, which suggests that the programme increased the sense of preparedness among graduates who did not initially feel ready for their role as an intern, but also decreased the sense of preparedness among some individuals who had initially rated themselves on the higher end of the scale. We interpret this finding as suggesting that a subset of interns may have demonstrated overconfidence in their knowledge and abilities prior to participation, and that this sense of preparedness was grounded when the programme provided context and perspective on the challenges they would face as an intern. This finding is especially important in light of much research on the risks of overconfidence in the practice of clinical medicine [31–33], and was an unexpected outcome of our programme.

### Creating a healthcare community

Another positive impact of the programme was in building a sense of community among incoming trainees and between trainees and their future supervisors. In a recent study conducted in two districts in Botswana, healthcare workers reported a sense of isolation and poor communication as factors limiting organizational effectiveness and outlined their desire for an organizational culture characterized by teamwork and shared vision [34]. More than 60 session leaders, panelists, and clinical instructors from across Botswana’s healthcare sectors participated and contributed to the programme – demonstrating the vitality of the country’s healthcare community and motivation of one generation of healthcare providers to pass their knowledge, experience, and values to the next. Programmes such as this may help to address these issues, and the impact of our programme related to this finding should be explored further.

### Botswana’s programme in context

Incoming interns in many settings have been shown to feel underprepared for their transition to serving as care providers [35], and the challenges associated with this particular transition have been well-described [24]. In response, pre-internship training programmes are gaining popularity worldwide, particularly in high-income country settings and in many cases preceding surgical training [36–38]. While most are based within a single institution (medical school or post-graduate medical programme), some countries have implemented national programmes [39], although we were unable to identify reports on similar programmes in resource-constrained or sub-Saharan African settings.

Pre-internship programmes are usually of limited duration (several hours to several weeks) and focus on the acquisition of discrete skills. Self-assessment is usually used in evaluation of these programmes [36, 40, 41], although some have implemented objective knowledge assessments [42]. While participants often demonstrate improved self-confidence [36], which has proven to be durable in some settings [40], other authors have not reported on the phenomenon of tempered self-confidence that we observed in a subset of our participants.

Overall, our programme was unique in the context in which it was implemented, expands upon the reported outcomes of similar programmes, particularly with regard to its possible impact on overconfidence and its encouragement of community building, and adds to the literature on such programmes that is lacking in reports from resource-constrained settings.

### Limitations

We report on the results of a novel programme, which was a two-week long pilot intervention prior to the initiation of a full year of internship training. Gathering interns’ retrospective feedback on the programme at a point further along their training may add valuable insight on the programme’s impact, which may not be represented in our current report. In addition, studying the impact of similar pre-internship programmes on future cohorts of medical graduates would be necessary to demonstrate a consistent and replicable impact. Importantly, our results must be interpreted with caution as they are based upon interns’ self-rated knowledge and skills, and we did not test these objectively. While self-assessment is a practical tool for evaluation, it is subjective, vulnerable to bias, and limited in its agreement with objective measurements of performance [43]. This method was chosen for this project because it was deemed to be the most acceptable form of evaluation to participants in the programme; obviated potential concerns that external assessment could affect the training prospects of programme participants; and was the most practical to implement in view of the programme’s setting and available resources.

### Lessons learned and questions raised

Reflecting on the practical implementation of this programme, we make the following observations:

1. The programme’s development and implementation required investment of time, attention, and funds on the part of existing offices and administrative structures within key stakeholder organizations and their partners. A collaborative effort was essential given the scope of the programme’s objectives, which would not have been possible to achieve by any one stakeholder in isolation.
2. Diversity in the professional backgrounds, affiliations, and career paths of programme faculty should be sought to familiarize participants with various aspects of the medical system and represent the full breadth of the medical community. In our setting, the enthusiastic involvement of contributors from across the local health system and medical community enhanced the overall quality of the programme and successfully engaged participants.
3. Programme and administrative coordinators were crucial for the development of a programme with a unified vision and to ensure its smooth implementation.
4. In general, participants considered programme content to be most useful when it was directly applicable to their future training and career and administered in interactive small group sessions. Future design of similar programs will require consideration of how to best implement the most effective formats and contents within the necessary constraints of time, setting, and available resources.
5. Careful consideration, at the programme planning stage, should be given to assessment methods that are practical to implement and would provide the most reliable appraisal of impact on trainees and attainment of stated objectives.

A number of questions remain. Most important is whether such a programme has an impact on objective outcomes of participant knowledge, skills, and attitudes, which would require a more robust assessment infrastructure and different appraisal metrics that would remain acceptable to the participants. Such formal objective assessment is likely to lead to some more questions: How would we manage a situation in which a serious knowledge or ability gap were identified in a participant during this programme, or one in which consistent limitations were observed in graduates of a particular medical school? These will be among the important considerations going forward.

Furthermore, in Botswana, the strengthening of medical training programmes is seen as a key element of a national strategy aimed at promoting doctor retention. This is the context in which MIT was created and in which the programme presented here was undertaken. Questions remain regarding the actual impact programmes like this can have on trainees’ eventual career paths and life choices. The positive impact demonstrated on participants’ sense of community suggests that such interventions may help increase trainees’ sense of belonging and commitment to the local medical system. Evaluation of MIT’s impact on the overall national goal of doctor retention should be the subject of further study.

## Conclusion

We report on a national programme to prepare medical school graduates for their transition into medical internship training in Botswana’s public healthcare system. We have demonstrated that the design and implementation of this type of programme is feasible, well-received by participants, and can have a positive impact on self-rated assessments of knowledge, skills, and attitudes despite its short duration. We believe the programme was successful in providing an organized transition from both in-country and foreign medical schools to structured internship training in Botswana: by improving graduates’ sense of preparedness and their familiarity with expected roles and responsibilities; by increasing their self-awareness with regard to their limitations; and by building a sense of community and camaraderie among this cohort of trainees. While generalizations about the feasibility and impact of this national programme should be made with caution, we hope this report will be useful to those working to strengthen medical training in health systems throughout sub-Saharan Africa or in similarly constrained settings worldwide.

## Additional file

Additional file 1: Curriculum plan for the programme. Detailed programme schedule with duration and content of each session. (PDF 113 kb)

## Abbreviation

UB: University of Botswana
MIT: Medical Internship Training Programme
