# Correction to: Strengthening medical training programmes by focusing on professional transitions: a national bridging programme to prepare medical school graduates for their role as medical interns in Botswana

**DOI:** 10.1186/s12909-018-1125-2

**Published:** 2018-02-12

**Authors:** Bunmi S. Malau-Aduli, Teresa O’Connor, Robin A. Ray, Yolanda van der Kruk, Michelle Bellingan, Peta-Ann Teague

**Affiliations:** College of Medicine and Dentistry, James Cook University, QLD, Townsville, Australia

## Correction

Following publication of the original article [1], one of the authors reported that prior to publication her surname had changed from ‘Kerlen’ to ‘van der Kruk’, but that this change had not been incorporated in the final version.

The original article has been updated.
