# Correction to: Strengthening medical training programmes by focusing on professional transitions: a national bridging programme to prepare medical school graduates for their role as medical interns in Botswana

**DOI:** 10.1186/s12909-018-1169-3

**Published:** 2018-03-27

**Authors:** Michael J. Peluso, Rebecca Luckett, Savara Mantzor, Alemayehu G. Bedada, Paul Saleeb, Miriam Haverkamp, Mosepele Mosepele, Cecil Haverkamp, Rosa Maoto, Detlef Prozesky, Neo Tapela, Oathokwa Nkomazana, Tomer Barak

**Affiliations:** 1 Botswana-Harvard Partnership, Gaborone, Botswana; 2 Department of Medicine and Division of Global Health Equity, Brigham and Women’s Hospital, Boston, MA USA; 3 Harvard Medical School, Boston, USA; 4 Department of Obstetrics and Gynecology, Scottish Livingstone Hospital, Molepolole, Botswana; 5 Department of Obstetrics and Gynecology, Beth Israel Deaconess Medical Center, Boston, USA; 6 Children’s Hospital of Philadelphia, Philadelphia, USA; 7 Department of Paediatrics, Princess Marina Hospital, Gaborone, Botswana; 8 Botswana-UPenn Partnership, Gaborone, Botswana; 9 Department of Surgery, Faculty of Medicine, University of Botswana, Gaborone, Botswana; 10 University of Maryland, College Park, MD USA; 11 Botswana-University of Maryland School of Medicine Health Initiative, Gaborone, Botswana; 12 Department of Medicine, Princess Marina Hospital, Gaborone, Botswana; 13 University Research Co, Gaborone, Botswana; 14 Medical Internship Training Programme, Gaborone, Botswana; 15 Department of Medical Education, University of Botswana, Gaborone, Botswana; 16 Botswana Ministry of Health, Gaborone, Botswana; 17 Faculty of Medicine, University of Botswana, Gaborone, Botswana; 18 Department of Medicine, Scottish Livingstone Hospital, Molepolole, Botswana; 19 Department of Medicine, Beth Israel Deaconess Medical Center, Boston, USA

## Correction

Following publication of the original article [1], the first author reported that there was a typographical error in the name of one of his co-authors. The correct spelling is Alemayehu Bedada, not Alemayhu Bedada.

The original article has been updated.
