# Supplementary material for: Strengthening medical training programmes by focusing on professional transitions: a national bridging programme to prepare medical school graduates for their role as medical interns in Botswana
Source: BMC Med Educ. 2017 Dec 21;17:261. doi: 10.1186/s12909-017-1102-1 (PMC5740920; doi:10.1186/s12909-017-1102-1)
Supplement: Additional file 1: — Curriculum plan for the programme. Detailed programme schedule with duration and content of each session. (PDF 113 kb) [file 12909_2017_1102_MOESM1_ESM.pdf]

Module
Module 1: The Botswana Healthcare Context
Module 2: Clinical Knowledge and Skills
Module 3: Becoming a Well-Rounded Clinician

			Agenda	
**Day/Session**	**Module**	**Content**	**Time Allocated**	**Topic**
Day 1 / AM	Module 1	Stakeholder Orientation	25 min	Welcome remarks
			25 min	Participant pre-programme self-assessment
			25 min	MIT key parameters
			25 min	Public service expectations
			25 min	The Botswana Health Professions Council
			25 min	Community perspectives
			25 min	Service-related standards and expectations
			25 min	Primary healthcare in Botswana
			25 min	Psychiatry in Botswana
			25 min	Guide to the healthcare system
Day 1 / PM	Module 2	Medical Emergencies Workshop Part 1	180 min	• Septic shock<br>• Hyperglycemic emergencies<br>• Unresponsive patient<br>• Chest pain
Day 2 / AM	Module 2	National HIV Training Day 1	180 min	HIV Training Part 1
Day 2 / PM	Module 2		180 min	HIV Training Part 2
Day 3 / AM	Module 2	National HIV Training Day 2	180 min	HIV Training Part 3
Day 3 / PM	Module 2		180 min	HIV Training Part 4
Day 4 / AM	Module 2	Surgical Skills Workshop Part 1	180 min	• Management of epistaxis<br>• Polytrauma
Day 4 / PM	Module 2	Surgical Skills Workshop Part 2	180 min	• Plaster application
Day 5 / AM	Module 3	Professionalism Workshop Part 1	180 min	• Introduction to professionalism<br>• Delivering bad news<br>• Managing “difficult” patients and families with compassion and understanding<br>• Coping in the workplace<br>• Hierarchy and whistle-blowing<br>• Transformative action and advocating positive change
Day 5 / PM	Module 3	Intern Perspectives	90 min	Tips from graduating interns
	Module 1	Career Paths	90 min	Panel and group discussion: “My professional future in Botswana”
Day 6 / AM	Module 2	Surgical Skills Workshop Part 3	180 min	• Evaluation and management of surgical wounds<br>• Suturing<br>• Knot tying
Day 6 / PM	Module 2	Surgical Skills Workshop Part 4	90 min	Ophthalmology Skills

Module 1: The Botswana Healthcare Context
Module 2: Clinical Knowledge and Skills
Module 3: Becoming a Well-Rounded Clinician

<table>
<tr><td>Day 7/ AM</td><td>Module 2</td><td>Medical Emergencies Workshop Part 2</td><td>180 min</td><td>• Seizure
• Hypertensive crisis
• Potassium disturbances
• Shortness of breath</td></tr>
<tr><td rowspan="2">Day 7 / PM</td><td rowspan="2">Module 3</td><td>Resources and Technology</td><td>90 min</td><td>Guidelines, apps, and accessing the medical literature</td></tr>
<tr><td>Professionalism Workshop Part 2</td><td>90 min</td><td>Panel and group discussion: Professionalism</td></tr>
<tr><td>Day 8 / AM</td><td>Module 2</td><td>OB/GYN Emergencies Workshop Part 1</td><td>180 min</td><td rowspan="2">• Bleeding in early pregnancy
• Antepartum hemorrhage
• Postpartum hemorrhage
Labor management
• Hypertensive disease in pregnancy
• Septic shock in OB/GYN</td></tr>
<tr><td>Day 8 / PM</td><td>Module 2</td><td>OB/GYN Emergencies Workshop Part 2</td><td>180 min</td></tr>
<tr><td>Day 9 / AM</td><td>Module 2</td><td>Pediatrics Emergencies Workshop Part 1</td><td>180 min</td><td rowspan="2">• Assessment of the critically ill child
• Respiratory distress
• Status epilepticus
• Shock in children
• Neonatal hypoglycemia
• Management of “flat” neonate
• Neonatal seizures
• Intraosseous line placement
• Pediatric intubation
• Neonatal resuscitation</td></tr>
<tr><td>Day 9 / PM</td><td>Module 2</td><td>Pediatrics Emergencies Workshop Part 2</td><td>180 min</td></tr>
<tr><td rowspan="8">Day 10 / AM</td><td rowspan="8">Module 1</td><td rowspan="4">Bridging Programme for Foreign Medical Graduates*</td><td>60 min</td><td>Tour of a national referral hospital</td></tr>
<tr><td>45 min</td><td>Tuberculosis in Botswana</td></tr>
<tr><td>30 min</td><td>Referral systems in Botswana</td></tr>
<tr><td>90 min</td><td>Group discussion: Transitional challenges</td></tr>
<tr><td rowspan="4">International Perspectives for In-Country Medical Graduates*</td><td>45 min</td><td>Medical schools and internship programs regionally and internationally</td></tr>
<tr><td>45 min</td><td>Botswana’s healthcare system an the international context</td></tr>
<tr><td>45 min</td><td>The South African healthcare system</td></tr>
<tr><td>45 min</td><td>Outcomes from Botswana’s medical school graduates</td></tr>
<tr><td rowspan="2">Day 10 / PM</td><td rowspan="2">Programme Conclusion</td><td>Assessment</td><td>30 min</td><td>Post-programme self-assessment</td></tr>
<tr><td>Parting words</td><td>30 min</td><td>Parting words</td></tr>
</table>

Note: In general, AM sessions ran from 9am to 12:30pm and PM sessions ran from 1:30pm to 5pm, with each session including a 30-minute break. *These sessions ran concurrently.
